# Warum brauchen wir Cardiac-Arrest-Zentren?

**DOI:** 10.1007/s00059-018-4728-9

**Published:** 2018-06-28

**Authors:** K. H. Scholz, B. W. Böttiger

**Affiliations:** 1 Klinik für Kardiologie und Internistische Intensivmedizin/Chest Pain Unit – Medizinische Klinik I, St. Bernward Krankenhaus GmbH, Treibestr. 9, 31134 Hildesheim, Deutschland; 2 Klinik für Anästhesiologie und Operative Intensivmedizin, Universitätsklinikum Köln (AöR), Kerpener Straße 62, 50937 Köln, Deutschland

**Keywords:** Herzstillstand, Myokardinfarkt, Reanimation, Katheterlabor, Koronarintervention, Heart arrest, Myocardial infarction, Resuscitation, Catheterization laboratory, Coronary intervention

## Abstract

Nach erfolgreicher prähospitaler Reanimation wird die weitere Prognose der betroffenen Patienten durch eine Reihe unterschiedlicher Faktoren beeinflusst. Prähospital hat die Dauer der Ischämie vom Zeitpunkt des Beginns des Kreislaufstillstands bis zum Beginn effektiver Reanimationsmaßnahmen die bei Weitem größte prognostische Bedeutung. Dieses Intervall kann v. a. durch eine Steigerung der Laienreanimationsquote verkürzt werden. Bezüglich der intrahospitalen Weiterversorgung hat eine Reihe von strukturellen Faktoren wie u. a. die Größe der Klinik und die Fallzahl der pro Jahr behandelten Postreanimationspatienten ebenfalls großen Einfluss auf die weitere Prognose. Ganz entscheidend sind dabei das Vorhandensein und die permanente Einsatzbereitschaft eines Herzkatheterlabors mit der Möglichkeit einer umgehenden Koronarintervention. Bei reanimierten STEMI(ST-Strecken-Hebungs-Myokardinfarkt)-Patienten hat der Zeitfaktor bis zur Wiedereröffnung des verschlossenen Infarktgefäßes eine ganz überragende Bedeutung für das Überleben. Eine 24/7-„Rund-um-die-Uhr“-Einsatzbereitschaft eines Katheterlabors zählt somit zu den unabdingbaren Voraussetzungen für ein Cardiac-Arrest-Zentrum. Daneben muss eine Reihe von technischen, strukturellen, medizinisch-inhaltlichen und organisatorischen Anforderungen in den Kliniken umgesetzt sein, um die Voraussetzungen für ein Cardiac-Arrest-Zentrum erfüllen zu können. Die Zertifizierung von Cardiac-Arrest-Zentren wird zurzeit durch den Deutschen Rat für Wiederbelebung (German Resuscitation Council, GRC) und die Deutsche Gesellschaft für Kardiologie (DGK) umgesetzt. Der GRC und die beteiligten Fachgesellschaften erhoffen sich hierdurch in erster Linie eine Vermeidung von Fehlzuweisungen von Postreanimationspatienten in zwar nächstgelegene, aber nicht spezialisierte und wenig oder nicht geeignete Kliniken. Künftige Analysen werden zeigen, wie sehr durch Cardiac-Arrest-Zentren die Prognose von erfolgreich prähospital reanimierten Patienten in Deutschland flächendeckend verbessert werden kann.

## Abstract (en)

In patients with out-of-hospital cardiac arrest (OHCA) and return of spontaneous circulation (ROSC) following cardiopulmonary resuscitation (CPR), the prognosis is influenced by various factors. In the prehospital setting, the duration of ischemia from the time of onset of cardiac arrest to the beginning of effective resuscitation measures is by far the most critical and determining factor for outcome. This interval can be shortened by an increase in the rate of lay CPR measures. With respect to intrahospital follow-up care, a number of structural factors have a relevant influence on prognosis. According to the literature, case volume, size of the hospital and the number of post-OHCA patients treated per year also have a large influence on the further prognosis. The crucial factor here is the availability and permanent readiness of a catheterization laboratory with the possibility of an immediate coronary intervention. In OHCA patients with ST-segment elevation myocardial infarction (STEMI), the time passed until the reopening of the occluded infarcted vessel is of paramount importance for survival. The 24/7 around the clock availability of a catheterization laboratory is therefore one of the indispensable prerequisites for a cardiac arrest center (CAC). In addition, a number of technical, structural, and organizational arrangements must be implemented in the CAC clinics in order to fulfil the requirements for such a center. The certification of CACs is currently being implemented by the German Resuscitation Council (GRC) and the German Society of Cardiology (DGK). As an important aim the GRC and the medical societies involved are hoping to avoid misallocation of post-OHCA patients to the nearest hospital, which may not be a suitable center for the treatment of these patients. Future studies will show whether CACs can indeed comprehensively improve the prognosis of OHCA patients following successful prehospital resuscitation.

## Einleitung

Die Erstpublikation der manuellen Thoraxkompression zur Überbrückung eines Herzkreislaufstillstands durch Kouwenhoven, Jude und Knickerbocker im Jahr 1960 [1] stellt aus heutiger Sicht einen Meilenstein in der Medizinhistorie dar. Das Verfahren wurde durch Peter Safar um die Mund-zu-Mund-Beatmung ergänzt, und im Jahre 1961 wurde auf einem Kongress in Baltimore erstmals das Konzept der kardiopulmonalen Reanimation vorgestellt [2].

In Deutschland werden aktuell pro Jahr prähospital rund 75.000 Reanimationen durchgeführt, dabei gelingt in etwa 40 % der Fälle die Wiederherstellung eines Spontankreislaufs („return of spontaneous circulation“, ROSC; [3]). Für die weitere Prognose der Patienten mit ROSC sind zwei Faktoren von wesentlicher Bedeutung, die durch systematische Eingriffe in Organisation, Prozessablauf und Struktur beeinflussbar sind:

- Ischämieintervall, d. h. die Zeitdauer vom Beginn des Kreislaufstillstands bis zum Beginn einer effektiven Thoraxkompression;
- Qualität und Spezialisierung der weiterbehandelnden Klinik.

Zur Verkürzung des Ischämieintervalls werden aktuell in verschiedenen Ansätzen und Projekten große Anstrengungen unternommen, wodurch auch bereits messbare Erfolge erreicht werden konnten. Zur Spezialisierung der Aufnahmekliniken, sog. Cardiac-Arrest-Zentren, wurden im letzten Jahr in einer Arbeitsgruppe des Deutschen Rates für Wiederbelebung (German Resuscitation Council, GRC) Kriterien zusammengestellt, die von den Fachgesellschaften der Anästhesiologen (DGAI), der Kardiologen (DGK) und der internistischen Intensivmediziner (DGIIN) konsentiert wurden [4]. Kliniken, die diese Kriterien erfüllen, sollen künftig als Cardiac-Arrest-Zentren zertifiziert werden.

## Verbesserung der Überlebensrate nach prähospitalem Kreislaufstillstand

### Verkürzung des Ischämieintervalls

Die Zeitdauer des sog. Ischämieintervalls von Beginn des Kreislaufstillstands bis zum Beginn einer effektiven Thoraxkompression sollte 3–5 min keinesfalls übersteigen, denn danach beginnt spätestens eine irreversible Hirnschädigung. In Deutschland trifft der professionelle Rettungsdienst in der Regel jedoch erst später am Notfallort ein, und es wäre eine Illusion, anzunehmen, dass innerhalb von 3–5 min bei einem relevanten Anteil der Patienten mit prähospitalem Kreislaufstillstand („out-of-hospital cardiac arrest“, OHCA) bereits professionelle Hilfe zur Verfügung steht.

Hier können allerdings Laien richtig helfen, und nach Literaturangaben kann bei OHCA die Überlebensrate durch Laienreanimation verdoppelt werden [5, 6]. Durch Öffentlichkeitsaktionen, wie z. B. der „Woche der Wiederbelebung“ und dem „European Restart a Heart Day“ konnte in den letzten Jahren der Anteil der Laienreanimationen in Deutschland und in Europa messbar gesteigert werden [7]. Ein effektives Mittel zur Steigerung der Quote der Laienreanimation ist die Telefonreanimation, d. h. die ganz gezielte Telefonanleitung von Zeugen eines Kreislaufstillstands zur Durchführung von Reanimationsmaßnahmen durch geschultes Personal der Rettungsleitstelle. Hier sind nach Literaturangaben lediglich wenige Telefonreanimationen nötig, um ein zusätzliches Leben zu retten [8, 9].

Noch größere Bedeutung zur Steigerung der Quote der Laienreanimation hat jedoch die – möglichst frühzeitige – Schulung breiter Bevölkerungsschichten (z. B. „KIDS-SAVE-LIVES“-Programm; [10]). In einigen europäischen Ländern konnte durch die Integration einer Reanimationsschulung in den Schulunterricht die Laienreanimationsquote erheblich gesteigert werden [11, 12]. Auch in Deutschland wurde inzwischen in einigen Bundesländern ein systematisches Reanimationstraining als Unterrichtsfach für Schüler eingeführt.

### Strukturbezogene Prädiktoren für die Überlebensrate nach OHCA

Nach Literaturangaben hat offenbar die reine Fallzahl der jährlich pro Klinik behandelten Patienten und besonders der Patienten mit OHCA prognostische Relevanz. Nach einer Untersuchung von Cha et al. lag bei Patienten, die unter Reanimationsbedingungen eingeliefert wurden, die Krankenhausüberlebensrate in „High-Volume-Zentren“ signifikant höher als in „Low-Volume-Zentren“ [13]. Auch Callaway und Mitarbeiter berichteten von einer Abhängigkeit der Prognose von der Fallzahl der in den Kliniken behandelten OHCA-Patienten. Nach dieser Untersuchung stiegen sowohl die Überlebensrate wie auch der Anteil der Patienten mit akzeptablem neurologischen Outcome etwa ab einer Zahl von 25 bis 30 jährlich pro Klinik behandelter Reanimationsfälle an [14]. Von der gleichen Autorengruppe war bereits zuvor beschrieben worden, dass bei Patienten mit prähospitaler Reanimation unabhängig von der Größe des Krankenhauses und der Anzahl der reinen Krankenhausbetten die Überlebensrate bei Verfügbarkeit eines Herzkatheterlabors signifikant höher lag [15]. Dies dürfte darin begründet sein, dass bei reanimierten Patienten der Anteil an Patienten mit koronarer Herzkrankheit besonders hoch ist. So liegt nach Literaturangaben bei rund 30 % der OHCA-Patienten ein akuter ST-Strecken-Hebungs-Infarkt („ST elevation myocardoial infarction“, STEMI) vor, und der Anteil der Nicht-ST-Strecken-Hebungs-Infarkte (NSTEMI) beträgt mindestens weitere 20–30 %. Insgesamt findet sich bei etwa 75–80 % der OHCA-Patienten letztlich eine kardiale Genese [16, 17].

### Prognostische Bedeutung einer frühen Koronarintervention nach OHCA

In einer Arbeit von Dumas und Mitarbeitern wurden in einem Kollektiv von 1001 Patienten, die nach OHCA lebend aus stationärer Behandlung entlassen werden konnten, mittels Propensity-Matching 46 Paare mit und ohne frühe perkutane koronare Intervention („percutaneous coronary intervention“, PCI) verglichen. Die Sterblichkeit im 5‑Jahres-Verlauf lag dabei in der PCI-Gruppe (*n* = 46 Patienten) mit 33 % signifikant niedriger als in der Gruppe ohne sofortige Koronarintervention (*n* = 46 Patienten) mit 67 % Todesfällen [18]. Eine Metaanalyse von 15 Studien mit insgesamt 3800 OHCA-Patienten ergab einen deutlichen Überlebensvorteil zugunsten der Akutangiographie mit einer Odds-Ratio (OR) von 2,77 (95 %-Konfidenzintervall [KI]: 2,06–3,72; [19]).

In frühen Daten aus dem Deutschen Reanimationsregister konnte bei 15.000 OHCA-Patienten gezeigt werden, dass der Koronarintervention und der Hypothermie offenbar eine große prognostische Bedeutung zukommt. Die Überlebensrate mit gutem neurologischen Ergebnis (CPC [„cerebral performance category“] 1 oder 2) war in diesem Beobachtungsregister bei reanimierten Patienten unter Normothermie bei durchgeführter Koronarintervention mit 23 % signifikant besser als ohne Koronarintervention mit lediglich 10 %. In dieser Analyse war die Prognose hingegen mit einer Überlebensrate von 49 % bei Weitem am besten, wenn die OHCA-Patienten sowohl mit PCI als auch mit Hypothermie behandelt worden waren [20]. Ähnliche Ergebnisse hat die Studie von Dumas und Mitarbeitern ergeben. Auch hier ging bei OHCA-Patienten die Kombination aus therapeutischer Hypothermie und PCI mit der signifikant besten Prognose einher [18]. Insgesamt zeigt sich – in der Gesamtschau aller Daten – eine Verdoppelung des Überlebens durch Behandlung in einem Zentrum mit der Möglichkeit zur sofortigen Koronarintervention.

**Abb. 1:**
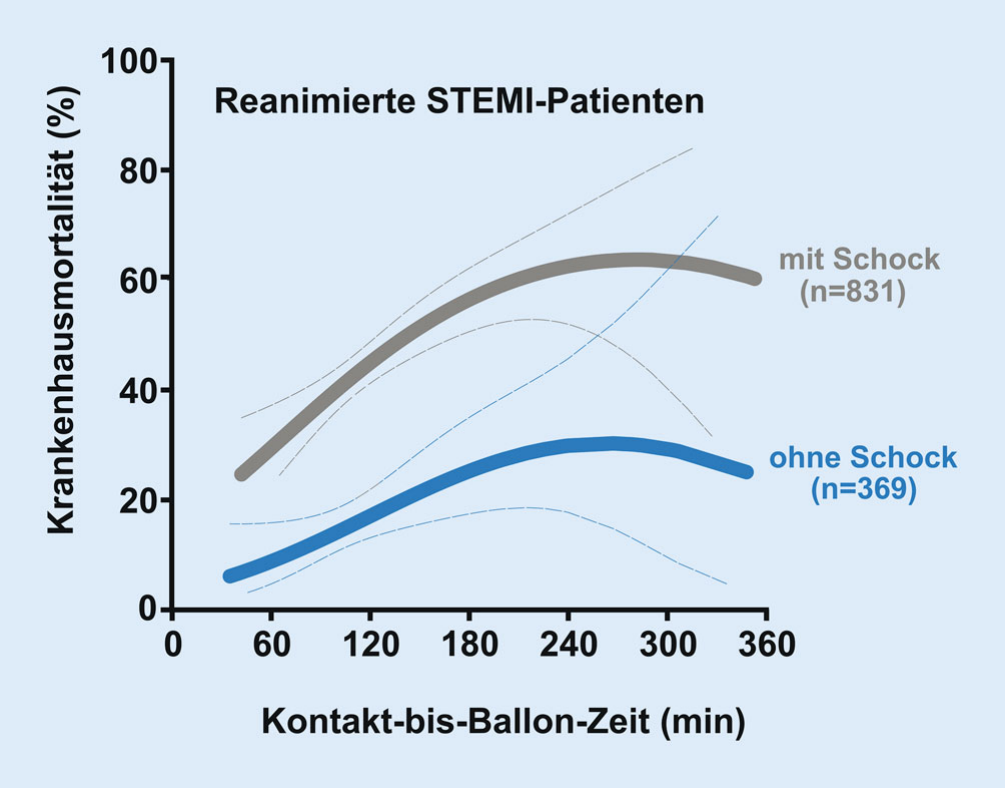
Abhängigkeit der Prognose von der Zeitdauer vom Erstkontakt mit dem Rettungsdienst bis zur Ballonwiedereröffnung des verschlossenen Kranzgefäßes (*Kontakt-bis-Ballon-Zeit*) bei Patienten mit OHCA („out-of-hospital cardiac arrest“) und STEMI („ST elevation myocardial infarction“; FITT-STEMI-Studie): Bei Kontakt-bis-Ballon-Zeiten zwischen 60 und 180 min erhöht sich die Sterblichkeit pro 10 min Zeitverlust in der OHCA-Gruppe mit Schock um 2,1 zusätzliche Todesfälle, in der OHCA-Gruppe ohne Schock um 1,3 zusätzliche Todesfälle, jeweils bezogen auf 100 behandelte Patienten. (Mod. nach [21])

Von ganz besonderer Bedeutung ist die unmittelbare Katheterintervention bei Patienten mit OHCA und akutem STEMI. Anhand von Daten der FITT-STEMI-Studie konnte kürzlich gezeigt werden, dass bei STEMI-Patienten mit OHCA und erfolgreicher prähospitaler Reanimation der Behandlungszeit bis zur Wiedereröffnung des Infarktgefäßes eine ganz entscheidende Rolle für die Prognose zukommt. In der Gruppe der Patienten mit PCI innerhalb von 90 min lag die Krankenhaussterblichkeit mit knapp 23 % wesentlich niedriger als mit gut 40 % bei OHCA-STEMI-Patienten mit einer Behandlungsdauer von mehr als 90 min [21]. An dem gleichen Kollektiv konnte gezeigt werden, dass die Beziehung zwischen der Zeitdauer nach Erstkontakt mit dem Rettungsdienst bis zur Rekanalisation des Infarktgefäßes und der Krankenhaussterblichkeit in den frühen Stunden nahezu linear ist: Bei STEMI-Patienten mit OHCA und bestehender Schocksituation bei Eintreffen in der Klinik kam es im Intervall zwischen 60 und 180 min nach medizinischem Erstkontakt pro 10 min Zeitverlust bis zur Wiedereröffnung des Infarktgefäßes zu jeweils zusätzlich 2,1 Todesfällen auf 100 behandelte Patienten und bei OHCA mit hämodynamisch stabiler Situation ohne Schock zu zusätzlich 1,3 Todesfällen pro jeweils 10 min Zeitverlust (Abb. 1; [21, 22]). Bei Patienten mit prähospitaler Reanimation muss somit durch den Rettungsdienst unmittelbar nach Erreichen von ROSC eine 12-Kanal-EKG-Registrierung erfolgen. Im Fall einer STEMI-Diagnose oder eines STEMI-Verdachts muss der Patient dann unter Umgehung der Nicht-PCI-Klinik (Kliniken, die keine „Rund-um-die-Uhr“-PCI-Möglichkeit anbieten) und möglichst auch unter Umgehung der Notaufnahme der PCI-Klinik direkt in ein funktionsbereites Herzkatheterlabor transportiert werden.

## Spezialisierung und Qualität der weiterbehandelnden Klinik: Cardiac-Arrest-Zentren

**Abb. 2:**
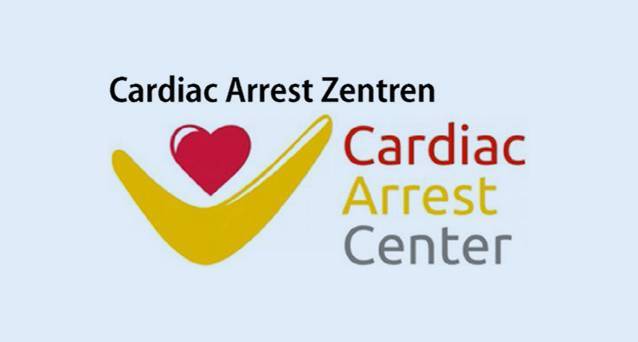
Cardiac-Arrest-Zentrums-Logo für die Zertifizierung der Zentren durch den Deutschen Rat für Wiederbelebung (German Resuscitation Council, GRC)

In einem Statement der American Heart Association (AHA) wurde bereits im Jahr 2010 die Einrichtung von Cardiac-Arrest-Zentren zur speziellen Weiterbehandlung von OHCA-Patienten vorgeschlagen [23]. Diese Empfehlung wurde soeben aktualisiert [24]. Auch in Deutschland ist die Gründung von Cardiac-Arrest-Zentren seit Jahren in Diskussion (Abb. 2 und 3). Das Thema wurde bei den Bad Boller Reanimationsgesprächen im Jahr 2014 weiter fokussiert [25], und die Etablierung von Cardiac-Arrest-Zentren wird auch in den aktuellen nationalen und internationalen Reanimationsleitlinien aus dem Jahr 2015 gefordert [26].

Zwischenzeitlich sind dann auch in Deutschland viele Cardiac-Arrest-Zentren selbsternannt entstanden. Darüber hinaus zeigen sich sehr viele Kliniken an dem Thema interessiert. In einer Arbeitsgruppe des GRC wurde in der Folge ein Kriterienkatalog erstellt, in dem die diesbezüglichen strukturellen und organisatorischen Anforderungen nach Expertenkonsens zusammengetragen wurden. Diese Qualitätsindikatoren und strukturellen Erfordernisse wurden zwischenzeitlich von DGAI, DGK und DGIIN konsentiert und im Jahr 2017 publiziert [4].

Zu den wesentlichen Kriterien für Cardiac-Arrest-Zentren gehört die Überprüfbarkeit einer standardisierten Postreanimationsbehandlung. Allgemeine Voraussetzungen hierfür sind das Vorhandensein spezieller Fachdisziplinen: Neben der interventionellen Kardiologie mit ständiger Rufbereitschaft für Katheterbehandlung bei STEMI sind dies die Anästhesiologie, die Neurologie und ggf. auch die Unfallchirurgie. Es wird das Vorhandensein eines nachweislich rund um die Uhr einsatzbereiten Herzkatheterlabors gefordert. Hinzu kommt die Notwendigkeit einer adäquaten technischen Ausrüstung wie z. B. Computertomographie (CT), Röntgen, Echokardiographie einschließlich transösophagealer Echokardiographie und moderner Beatmungs- und Dialyseausstattung. Darüber hinaus sollen standardisierte Behandlungspfade existieren, z. B. für Patienten mit STEMI oder Traumapatienten. Auch sollen die zeitlichen Abläufe in der Postreanimationsversorgung standardisiert protokolliert werden. Absolut unabdingbar sind die Verfügbarkeit einer rund um die Uhr bestehenden Herzkatheterbereitschaft sowie die Ausstattung zu leitliniengerechtem Temperaturmanagement.

Nach den Kriterien sollen auch SOP („standard operating procedures“) für unterschiedliche Prozesse verfügbar sein, unter anderem zur Übernahme von Patienten nach prähospitaler Reanimation wie auch nach intrahospital durchgeführter Reanimation durch ein definiertes Cardiac-Arrest-Team. Auch werden SOP zur Kommunikation zwischen den Rettungsdiensten und dem Cardiac-Arrest-Zentrum verlangt. Dies bezieht sich auf die Dokumentation der Notfallanmeldung, auf die Definition von Kommunikationswegen und auf die Festlegung von Verantwortlichkeiten. Es sollen eine SOP zur Notfalldiagnostik bei Aufnahme nach Reanimation zur Intensivtherapie einschließlich Temperaturmanagement und eine SOP zum strukturierten Outcome-Assessment (u. a. auch Therapieabbruch – hier wurde nach aktuellen Analysen in der Vergangenheit wahrscheinlich viel zu häufig zu früh eine Therapie eingestellt [24]) bestehen.

Darüber hinaus sollten der Behandlungsverlauf und auch das Outcome der Patienten möglichst auch im weiteren Follow-up nach 30 Tagen und einem Jahr erfasst werden. Eine solche Datenerfassung kann beispielsweise im deutschen Reanimationsregister der Anästhesiologen, aber speziell auch in einem Register des FITT-STEMI-Projekts durchgeführt werden. Empfohlen wird darüber hinaus ein systematisches Feedback mit der gesamten Rettungs- und Therapiekette in gewissen zeitlichen Abständen, so wie es im FITT-STEMI-Projekt bereits seit Jahren erfolgreich umgesetzt wird [27]. Dafür sollten in den Kliniken Qualitätszirkel implementiert werden. Auf den Boden dieser Erfordernisse kann dann eine Zertifizierung solcher Zentren durch den GRC in Kooperation mit der DGK und ggf. anderen Fachdisziplinen umgesetzt werden.

**Abb. 3:**
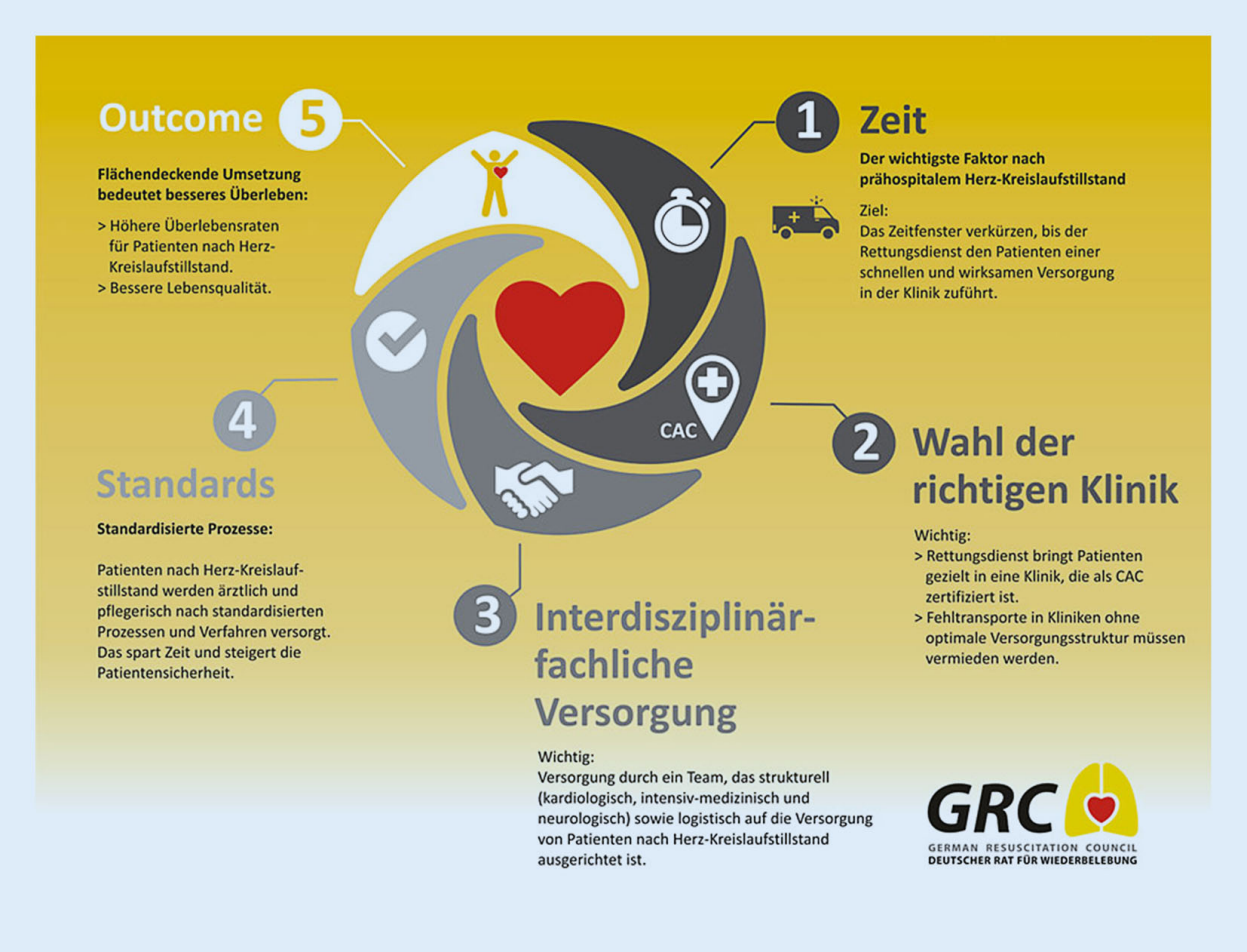
CAC(„cardiac arrest center“)-Infografik des Deutschen Rates für Wiederbelebung (German Resuscitation Council, GRC)

## Führen Cardiac-Arrest-Zentren tatsächlich zu einer Verbesserung der Behandlungsqualität bei Patienten mit OHCA, und warum brauchen wir das wirklich?

Zusammengefasst gibt es derzeit eine Reihe von ganz wesentlichen und eindeutigen Hinweisen darauf, dass gewisse strukturelle Faktoren, wie z. B. die Größe und die Fallzahl einer Klinik, die Anzahl der jährlich pro Klinik behandelten Patienten mit OHCA, das Vorhandensein eines einsatzbereiten Herzkatheterlabors sowie die technische Ausstattung jeweils einen sehr relevanten Einfluss auf die Qualität der Postreanimationsbehandlung und auf die Prognose bei OHCA-Patienten haben. Elmer und Mitarbeiter konnten kürzlich ein signifikant besseres Langzeit-Outcome an einem High-Volume-Cardiac-Arrest-Zentrum nachweisen im direkten Vergleich zu 6 anderen weniger oder nicht spezialisierten Kliniken [28]. Den direkten Beweis für eine Prognoseverbesserung durch die Einrichtung von Cardiac-Arrest-Zentren *per se* – in Form von großen randomisierten, kontrollierten und verblindeten Studien – gibt es derzeit jedoch nicht. Ein solcher Beweis kann erst geführt werden, wenn bei einer großen Anzahl von existierenden Cardiac-Arrest-Zentren die Outcome-Daten bei OHCA-Patienten systematisch risikoadjustiert mit den Ergebnissen anderer Kliniken ohne Cardiac-Arrest-Zentrum verglichen werden können. Es gibt jedoch bereits jetzt sehr klare Ergebnisse, dass das Vorhandensein einer „Rund-um-die-Uhr“-PCI-Einsatzbereitschaft bei Patienten nach OHCA prognosebestimmend ist und dass ganz besonders bei OHCA-Patienten mit STEMI der Zeitfaktor enormen Einfluss auf die Sterblichkeit hat.

## Fazit für die Praxis

- Für die Kliniken bedeutet die Etablierung als Cardiac-Arrest-Zentrum die Möglichkeit, sich den Rettungsdiensten als professionelles Glied in der Rettungs- und Therapiekette nach erfolgreicher prähospitaler Reanimation anzubieten.
- Für die Rettungsdienste bedeutet dies die Möglichkeit einer zielsicheren Zuweisung betroffener Patienten in überprüfbar spezialisierte Krankenhäuser. Ein ganz wesentlicher Aspekt ist, dass dadurch Fehltransporte in Kliniken, die diese Anforderungen nicht erfüllen können, durch die Rettungsdienste vermieden werden, was für die betroffenen Patienten von erheblicher prognostischer Bedeutung bezüglich des Überlebens ist. Allein dadurch erscheint der Aufwand einer Etablierung und Zertifizierung von Cardiac-Arrest-Zentren bereits gerechtfertigt.
- Der GRC erwartet – neben der Verbesserung des Überlebens – von der bundesweiten Umsetzung des Konzepts der Cardiac-Arrest-Zentren und deren Zertifizierung darüber hinaus im Wesentlichen folgende Impulse für die Optimierung der Postreanimationshandlung:
  - einheitliche Struktur der Prozessabläufe an den Schnittstellen Präklinik und Klinik in den entsprechenden Zentren,
  - maximale Professionalisierung und Optimierung der weiteren Abläufe in den Kliniken,
  - flächendeckende Umsetzung der Kriterien an möglichst allen hierfür in Frage kommenden Kliniken und somit eine bestmögliche Postreanimationsversorgung flächendeckend in ganz Deutschland.
- Eine solche Aufgabe kann als Projekt nur gemeinsam mit den Leitstellen, den Rettungsdiensten und den Kliniken sowie den beteiligten Fachgesellschaften gemeistert werden.
